# Effects of the Addition of Co or Ni Atoms on Structure and Magnetism of FeB Amorphous Alloy: Ab Initio Molecular Dynamics Simulation

**DOI:** 10.3390/ma14216283

**Published:** 2021-10-21

**Authors:** Shuwei Lu, Lei Xu, Biaobing Cao, Haiming Duan, Jun Zhang, Qiang Li

**Affiliations:** 1 Xinjiang Key Laboratory of Solid State Physics and Devices, Xinjiang University, Urumqi 830046, China; lushuwei@xju.edu.cn (S.L.); xulei@xju.edu.cn (L.X.); bbcao@xju.edu.cn (B.C.); 2 School of Physical Science and Technology, Xinjiang University, Urumqi 830046, China

**Keywords:** amorphous alloys, ab initio molecular dynamics, magnetic properties

## Abstract

The effects of the substitution of Fe by Co or Ni on both the structure and the magnetic properties of FeB amorphous alloy were investigated using first-principle molecular dynamics. The pair distribution function, Voronoi polyhedra, and density of states of Fe_80−x_TM_x_B_20_ (x = 0, 10, 20, 30, and 40 at.%, TM(Transition Metal): Co, Ni) amorphous alloys were calculated. The results show that with the increase in Co content, the saturation magnetization of Fe_80−x_Co_x_B_20_ (x = 0, 10, 20, 30, and 40 at.%) amorphous alloys initially increases and then decreases upon reaching the maximum at x = 10 at.%, while for Fe_80−x_Ni_x_B_20_ (x = 0, 10, 20, 30, and 40 at.%), the saturation magnetization decreases monotonously with the increase in Ni content. Accordingly, for the two kinds of amorphous alloys, the obtained simulation results on the variation trends of the saturation magnetization with the change in alloy composition are in good agreement with the experimental observation. Furthermore, the relative maximum magnetic moment was recorded for Fe_70_Co_10_B_20_ amorphous alloy, due to the induced increased magnetic moments of the Fe atoms surrounding the Co atom in the case of low Co dopant, as well as the increase in the exchange splitting energy caused by the enhancement of local atomic symmetry.

## 1. Introduction

Amorphous alloys are well-known to exhibit excellent mechanical and magnetic properties due to their short-range order, non-grain boundary and isotropic structure. Since the successful preparation of the Au_80_Si_20_ amorphous alloy by Paul Duwez through rapid solidification in 1960 [1], exploration of the structure and properties of amorphous alloys has attracted substantial interest among researchers, especially in regard to the investigation of the magnetic properties of the amorphous alloys. In 1967, the amorphous ferromagnetic alloy Fe_80_P_13_C_7_ was first prepared and was characterized by its disordered structure and the coexistence of a wide range of magnetic orders [2]. The development of Fe-based amorphous alloys has drawn valuable attention due to their minimal core loss, low coercivity and remanence, high permeability, and low costs. In fact, it is due to these properties that Fe-based amorphous alloys are now used in transformer cores, bringing more advantages over the traditional silicon steel. Due to these advantages, Fe-based amorphous alloys are used in various important applications. Nowadays, several research groups adopt various methods to investigate the magnetic properties of amorphous alloys. Through the improvement of preparation technology, researchers can synthesize excellent magnetic amorphous alloys [3,4,5,6]. Amorphous alloys of larger size with ferromagnetic properties can also be prepared by component design [7,8]. To date, several important amorphous alloys with excellent magnetic properties have been developed and widely used, such as magnetic shielding doors, sensors, anti-theft devices, cable shielding, and magneto-optical recording, among others [9,10,11]. The continuous advancement of related industries will inevitably lead to further extensive exploration and research on the magnetic properties of amorphous materials.

The magnetic theory of amorphous alloys has been developed with the study of amorphous magnetism. Similar to the investigation of the magnetic properties of crystalline alloys, there are two main competing theories in the study of the magnetic properties of amorphous alloys, namely, the local electron theory and the itinerant electron theory. Based on the local electron theory, Corb et al. developed a coordination bond model [12] to explain the magnetic properties of crystalline and amorphous alloys, which is derived from the concept of local magnetic moments. The model focused on local atomic arrangements and, especially, on the coordination around metalloid atoms, showing that the decrease in the magnetic moments of the alloy is to be attributed to the effect of chemical bonds. From the point of view of the itinerant electron theory, Williams et al. [13] used the band-gap theory to explain the magnetic properties of crystalline and amorphous alloys. It was found that the decrease in magnetism is mainly determined by the valence of the solute, independent of the local environments. However, these two models were found to show a bias in interpreting the experimental results [14,15] and there are currently no available theories and models that can fully explain the magnetic properties of all amorphous alloys.

The most significant foundation of many studies on alloy magnetism is the discovery of the Slater–Pauling curve [16], which describes the relationship between the effective atomic magnetic moment of binary ferromagnetic alloys of Fe, Co, Ni, and other 3*d* transition elements and the average number of 3*d* + 4*s* electrons present in an atom of the alloy. When Ni or Co forms an alloy with Fe, the resulting alloy exhibits a different set of magnetic properties. The incremental addition of Ni results in the continuous decrease in the magnetic moment of the alloy. However, when Co is added to Fe, the magnetic moment increases initially and, eventually, decreases after reaching a maximum magnetic moment [17,18]. Similar findings have been reported for corresponding amorphous alloys [19,20]. As amorphous alloys have the characteristics of short-range order and long-range disorder, most explanations of how the addition of Ni or Co to Fe-based amorphous alloys affects the magnetic moment directly are applied to the interpretation of the corresponding alloy magnetic theory. However, although the theory is sufficient to discuss the observed phenomena, some findings are also found to deviate. Therefore, the mechanism of substituting Fe with Ni or Co and its effects on the saturation magnetization (*Ms*) of Fe-based amorphous alloys is still unclear. To date, in order to better understand the structure and properties of amorphous materials, computer simulations have been used as an effective auxiliary means of experimental observation. The computational approach provides an effective way to explore the geometric structure, electronic structure, and internal correlation of crystal state and amorphous materials at an atomic scale [21,22,23,24,25,26,27]. In this work, the atomic structure and electronic properties of Fe_80-x_TM_x_B_20_ (x= 0, 10, 20, 30, and 40 at.%, TM: Co, Ni) amorphous alloys are systematically studied by employing a series of first principles molecular dynamics simulation. The effects of replacing Fe with either Ni or Co on the *M_S_* of the alloy are discussed. The intrinsic relationship between local atomic structure and magnetism of Fe-based amorphous alloys is also analyzed. 

## 2. Calculation Method

Fe_80−x_TM_x_B_20_ (x = 0, 10, 20, 30, and 40 at.%; TM: Co, Ni) amorphous alloys were studied by means of the Vienna ab initio simulation package (VASP) software (VASP.5.4.4, University of Vienna, Wien, Austria) based on the density functional theory [28]. The Perdew–Wang generalized gradient approximation exchange-correlation functional was adopted alongside the projected augmented wave method [29,30]. A canonical ensemble was applied at each temperature step and the temperature was controlled using a nose thermostat [31]. Due to the lack of symmetry in the amorphous alloys, the Brillion zone was sampled using only the Г point. The dimensions of a cubic supercell containing 200 atoms under periodic boundary conditions were inferred based on the experimentally obtained density of the amorphous alloy. The initial structure was constructed by randomly distributing atoms in a cubic box. The Verlet algorithm was used to solve Newton’s equation and the time step was set to 3 fs. The alloy was quenched to 300 K at a cooling rate of 1.67 × 10^14^ K/s starting from the melting state at 1600 K and the amorphous structure was thus obtained. Total pair distribution functions (TPDFs), partial pair distribution functions (PPDFs), and Voronoi polyhedra (VPs) [32] were calculated from the ensemble statistics of 1000 configurations. The study of individual atoms was based on the final configuration of the structure using a Bader partition [33], which was considered to be the outline of the minimum charge density at the atomic boundaries. In addition, the magnetic properties are discussed on the basis of the spin-polarized density of states (DOS) and partial density of states (PDOS).

## 3. Results and Discussions

**Figure 1:**
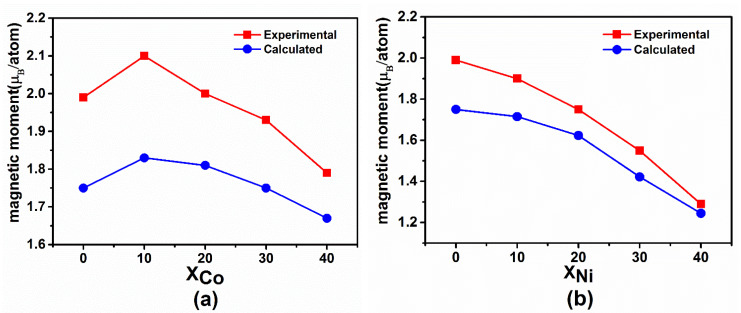
Comparison of magnetic moments of (**a**) Fe_80−x_Co_x_B_20_ and (**b**) Fe_80−x_Ni_x_B_20_ (x = 0, 10, 20, 30, and 40 at.%) amorphous alloys obtained via experiments and calculations.

Figure 1 shows the magnetic moments of Fe_80−x_Co_x_B_20_ and Fe_80−x_Ni_x_B_20_ (x = 0, 10, 20, 30, and 40 at.%) amorphous alloys obtained from the experiments [19] and theoretical simulations. The saturated magnetic moment of Fe_80−x_Co_x_B_20_ (x = 0, 10, 20, 30, and 40 at.%) amorphous alloys is shown in Figure 1a. For x < 10 at.%, the saturated magnetic moment increases with the Co content and the maximum *Ms* is achieved for a composition of Co of 10 at.%. For x > 10 at.%, the saturated magnetic moment decreases gradually. As can be seen from Figure 1b, the saturated magnetic moment of Fe_80−x_Ni_x_B_20_ (x = 0, 10, 20, 30, and 40 at.%) amorphous alloys gradually decreases with the increase in Ni content. The calculated results of the magnetic moment of the two amorphous alloys are in good agreement with the experimental results, which indicates the validity of the simulations. As the magnetic moments of Co and Ni are significantly smaller than those of Fe atoms, when the content of Co or Ni is high, the moment decreases quickly due to the dilution effect. Therefore, the doping concentration of Co and Ni was chosen to be only 40%. However, a small amount of Co is added, the magnetic moment increases. By contrast, the magnetic moment decreases monotonically upon adding Ni. The reasons for these phenomena are described in detail in the following sections.

### 3.1. Effects of the Local Geometric Symmetry on the Magnetism of the FeCoB Amorphous Alloys

**Figure 2:**
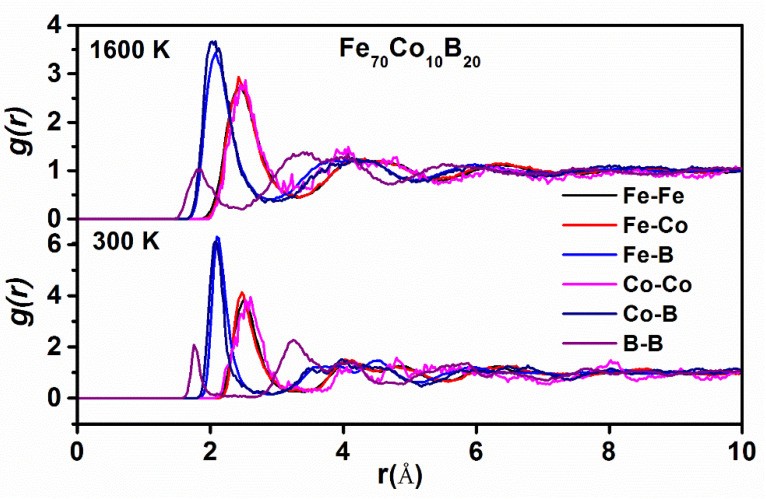
Partial pair distribution functions (PPDFs) at 1600 K and 300 K in Fe_70_Co_10_B_20_ amorphous alloys.

Although amorphous alloy is a long-range disordered system, there still exists a short-range ordered structure. Local atomic structure of each amorphous alloy was analyzed by using PPDFs. Here, Fe_70_Co_10_B_20_ alloys were chosen for analysis. The PPDFs g(r) of TM–TM, TM–B (TM: Fe, Co) and B–B in Fe_70_Co_10_B_20_ alloys at 1600 K and 300 K are shown in Figure 2. The height of first peak of the PPDFs at 300 K is higher than that at 1600 K. Most of the peaks shift toward larger distance, and there is a split in the second peak of PPDF, which is a typical amorphous feature. It shows that short-range order becomes the dominant with the decrease in temperature. However, the first peak of $g_{B-B}\left(r\right)$ is relatively low at 300 K, indicating that there is direct contact between solute and solute. Therefore, there exists a B–B bond. It also can be found that $g_{\mathrm{TM}-B}\left(r\right)$ has a higher intensity compared with the $g_{\mathrm{TM}-\mathrm{TM}}\left(r\right)$ at 300 K. There is a strong interaction between Fe or Co atoms and metalloid B atoms. Meanwhile, when the temperature changes from 1600 K to 300 K, position of the first peak of $g_{\mathrm{Fe}-\mathrm{Fe}}\left(r\right)$ or $g_{\mathrm{Fe}-\mathrm{Co}}\left(r\right)$ gradually moves to a larger distance, indicating that the bond length gradually increases, which is attributed to the transition of amorphous alloys from paramagnetism to ferromagnetism.

**Figure 3:**
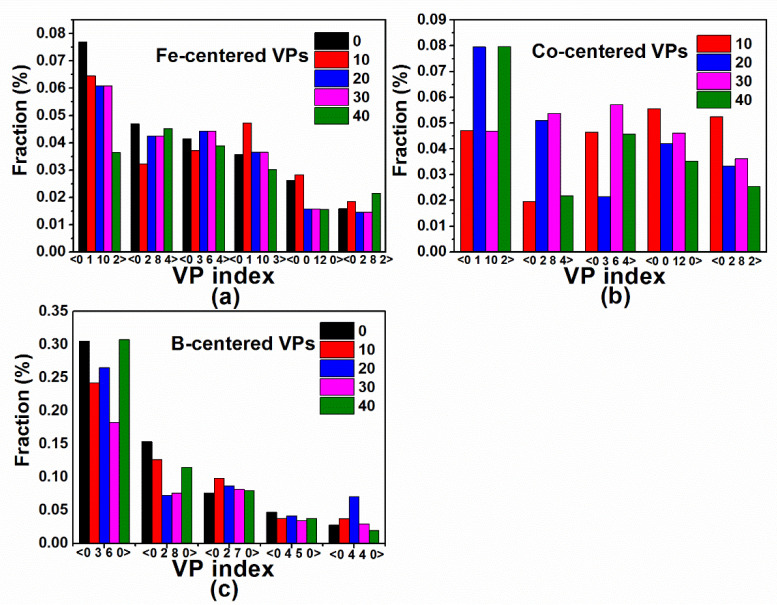
Numbers of Voronoi polyhedral (VPs) with central (**a**) Fe, (**b**) Co, and (**c**) B atoms in Fe_80−x_Co_x_B_20_ (x = 0, 10, 20, 30, and 40 at.%) amorphous alloys at 300 K. VP index is expressed as $\langle n_{3}\;n_{4\;}n_{5\;}n_{6}\rangle$, where $n_{i}$ denotes the number of the i-edged faces of Voronoi polyhedron.

In order to understand the increase in the magnetic moment of Fe_80−x_Co_x_B_20_ (x = 0, 10, 20, 30, and 40 at.%) amorphous alloys after adding a small amount of Co atoms, the atomic packing characteristics were investigated via the VP analysis. The VPs of the five amorphous alloys were studied at 300 K. The VP index is expressed as $\langle n_{3}\;n_{4\;}n_{5\;}n_{6}\rangle$, where $n_{i}$ denotes the number of the i-edged faces of Voronoi polyhedron. The total number of the faces of the polyhedron corresponds to the coordination number of its center atom. Figure 3a–c show the predominant VPs with different atom centers for the five alloys. The VPs centered on Fe predominantly consist of twisted icosahedra centered on <0 1 10 2>, <0 2 8 4>, <0 3 6 4>, <0 1 10 3>, and <0 2 8 2>, but a perfect icosahedron centered on <0 0 12 0> can also be observed. Co-centered VPs mainly include <0 1 10 2>, <0 2 8 4>, <0 3 6 4>, and <0 2 8 2> twisted icosahedral clusters, as well as some perfect <0 0 12 0 > icosahedron structures, indicating the position of the replacement of the Fe atom with the Co atom. The coordination numbers of the VPs centered on Fe or Co are about 13–14. The B-centered VPs are mainly shown as <0 3 6 0> (tri-capped trigonal prisms (TTPs)), <0 4 4 0> (Archimedean antiprisms), and <0 2 8 0> icosahedral-like clusters. The coordination number of the VPs with B as the center atom is mostly in a range between 8 and 9. It is also found that, for a Co content of 10%, the number of perfect icosahedral structures centered on Fe and Co atoms is the largest compared with that of other alloy components, indicating that the addition of Co increases the symmetry of the local structure of the Fe_70_Co_10_B_20_ amorphous alloy.

**Figure 4:**
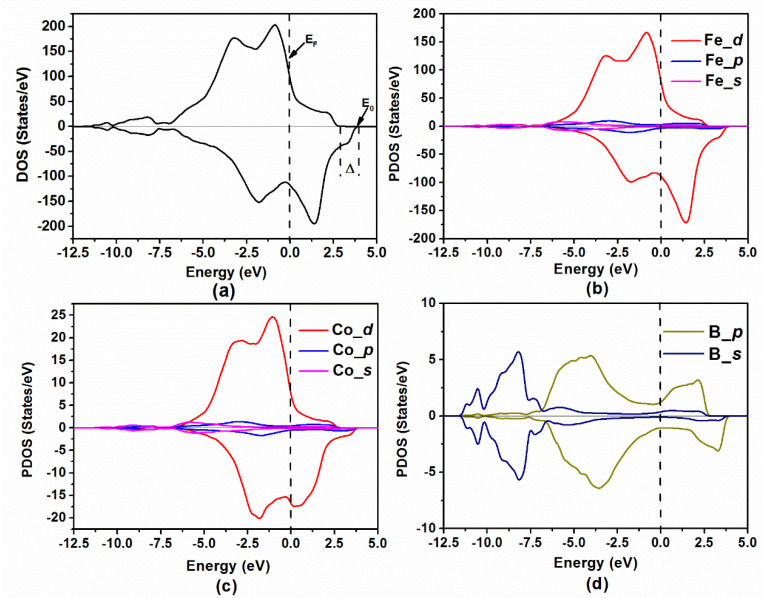
(**a**) Total density of states (DOS) of Fe_70_Co_10_B_20_ amorphous alloy, where Δ is exchange splitting energy, E_F_ is Fermi level, and E_0_ is the top of the energy band. (**b**–**d**) partial density of states (PDOS) of Fe_70_Co_10_B_20_ amorphous alloy for Fe, Co, and B, respectively.

In addition, the spin-polarized DOS of Fe_80−x_Co_x_B_20_ (x = 0, 10, 20, 30, and 40 at.%) amorphous alloys was calculated based on the equilibrium structure. As the DOSs of the five amorphous alloy states are similar, the Fe_70_Co_10_B_20_ amorphous alloy was selected for the analysis. Figure 4a shows that the amorphous alloy is a weak ferromagnet, in which both spin bands are partially filled and the exchange splitting energy (Δ) is smaller than the energy difference between the Fermi level (E_F_) and the top of the energy band (E_0_). Similarly, the other four alloys are also weakly ferromagnetic. The PDOS of Fe and Co are shown in Figure 4b,c, respectively, which demonstrates that near the Fermi level, the electron states of Fe_70_Co_10_B_20_ amorphous alloy are dominated by 3*d* states of Fe and Co. Moreover, the contribution of the *p* states of B to the magnetic properties of the alloy is very small, as illustrated in Figure 4d. Notably, the *p* states of B overlap with the 3*d* states of Fe and Co, i.e., there is hybridization between them. The exchange splitting energies of Fe_80−x_Co_x_B_20_ (x = 0, 10, 20, 30, and 40 at.%) amorphous alloys were estimated to be 1.117, 1.127, 1.005, 0.921, and 0.851 eV, respectively. The splitting energy of the Fe_70_Co_10_B_20_ amorphous alloy is the highest, which may be due to the high local symmetry of this alloy. The number of perfect icosahedra in the Fe_70_Co_10_B_20_ alloy is higher than that in other alloys, and the icosahedron structure has a higher symmetry. This higher degree of symmetry results in an increase in the local magnetic moment [34,35], which leads to an increase in the splitting energy [36].

As the crystalline FeCo alloy has translational symmetry, however, for amorphous alloys, the fivefold symmetry is an important feature of the amorphous structure [37]. The perfect icosahedral structure in the fivefold symmetry has higher symmetry and the existence of more perfect icosahedrons may be one reason for the increase in the magnetic moment. 

### 3.2. Influence of the Charge Distribution on the Magnetism of the FeCoB and FeNiB Amorphous Alloys

**Figure 5:**
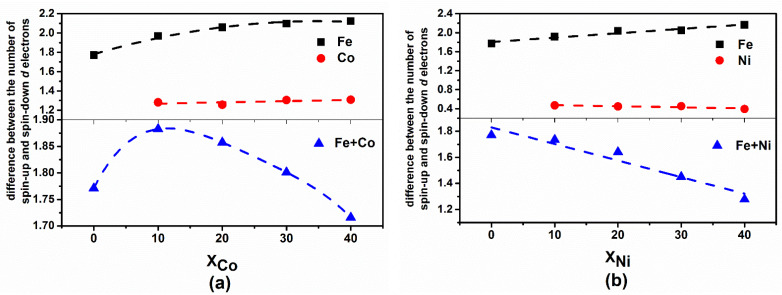
Difference between the number of spin-up and spin-down *d* electrons of (**a**) Fe_80−x_Co_x_B_20_ and (**b**) Fe_80−x_Ni_x_B_20_ (x = 0, 10, 20, 30, and 40 at.%) amorphous alloys. The dashed lines represent the fitted curves.

The magnetic behavior is mainly determined by the density and spin polarization of the electrons. To be short-range order and a long-range disorder, the structure of the amorphous alloy certainly affects the behavior of the electrons. This disordered potential field renders the electron wavefunction partially localized, so that the electrons are not uniformly distributed between the atoms. Table 1 lists the average values of the valence electrons of various elements in Fe_80−x_Co_x_B_20_ and Fe_80−x_Ni_x_B_20_ (x = 0, 10, 20, 30, and 40 at.%) alloys. Regarding the average gain and loss of electrons, the Fe atoms in Fe_80−x_Co_x_B_20_ or Fe_80−x_Ni_x_B_20_ (x = 0, 10, 20, 30, and 40 at.%) amorphous alloys tend to loose electrons and have a positive charge, while Co or Ni atoms tend to gain electrons on average. Moreover, B atoms prefer to gain electrons, which is consistent with the electronegativity of the B element. However, with the increase in Co or Ni atoms, the average electron loss of Fe atoms in both types of amorphous alloy increases gradually. The number of lost electrons is essentially related to the increase in the magnetic moment of the iron atom. As shown in Figure 5a,b. For Fe atoms, the difference between the number of spin-up and spin-down *d* electrons of the two amorphous alloys gradually increases, and the magnetic moment correspondingly increases. For Co or Ni atoms, the difference between the number of spin-up and spin-down *d* electrons hardly changes, indicating that the magnetic moment of Co or Ni atoms exhibits minor changes.

The sum of the difference between the number of spin-up and spin-down *d* electrons of Fe and Co (Ni) atoms was calculated, and the obtained trend is consistent with the change in the magnetic moment (Figure 1). Therefore, the larger the number of electrons that Fe loses, the greater the magnetic moment is; this is consistent with the Bader analysis. Similarly, the difference between the gain and loss of Co or Ni atoms at each investigated composition is small; thus, the magnetic moment change is also small. For Fe_80−x_Co_x_B_20_ (x = 0, 10, 20, 30, and 40 at.%) alloys, in the case of a small Co content, the magnetic moment of the alloy increases at first; however, as the Co content increases, the total magnetic moment of the alloy is gradually diluted, and thus decreases. Due to the dilution effect, the magnetic moment of Fe_80−x_Ni_x_B_20_ (x = 0, 10, 20, 30, and 40 at.%) alloys decreases gradually with the increase in the Ni content, as the magnetic moment of Ni is much smaller than that of Fe.

### 3.3. Effects of Neighboring Atoms on the Magnetism of the FeCoB and FeNiB Amorphous Alloys

**Figure 6:**
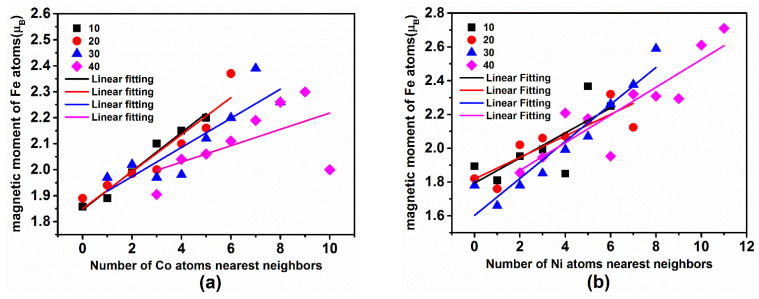
Magnetic moment of Fe atoms for different numbers of Co or Ni nearest neighbors in (**a**) Fe_80−x_Co_x_B_20_ or (**b**) Fe_80−x_Ni_x_B_20_ (x = 10, 20, 30, and 40 at.%) amorphous alloys. Solid lines in different colors indicate linear fitting curve.

Based on the above analysis, it was found that, with the increase in the Co or Ni content, the magnetic moment of Co or Ni atoms change little, while the magnetic moment of Fe atoms gradually increases. The reasons behind this behavior can be further analyzed from the perspective of the interaction between the neighboring atoms. Amorphous alloys do not exhibit the periodicity as crystalline solids, which are characterized by different local environments for each atom due to structure fluctuations and chemical short-order arrangement. As for the amorphous alloys investigated above, the distribution of the magnetic moment of the Fe atom is bound to be affected by the increase in the Co or Ni atom concentration. The relationship between the number of Co or Ni atoms near Fe and the average magnetic moment of the Fe atoms were determined. The four lines shown in Figure 6a,b are fitting curves, which represent an approximately linear relationship between the mean magnetic moment of Fe atoms for different Co or Ni content and the number of nearby Co or Ni atoms in the two types of amorphous alloy. As the Co or Ni concentration increases, the two types of amorphous alloy follow the same trend and the average magnetic moment of the Fe atoms increases with the number of nearby Co or Ni atoms.

For Fe_80−x_Co_x_B_20_ (x = 10, 20, 30, and 40 at.%) amorphous alloys, the number of Co atoms near each Fe atom gradually increases upon the increase in the Co concentration, and the magnetic moment of the Fe atom increases slowly and finally reaches saturation. For Fe_80−x_Ni_x_B_20_ (x = 10, 20, 30, and 40 at.%) amorphous alloys, the number of Ni neighboring atoms increases with the Ni concentration, finally reaching saturation. The reasons for this phenomenon can be analyzed from the interaction point of view. The magnetic properties of amorphous alloys are mainly determined by magnetic atoms. The interaction is modified by increasing the number of Co or Ni atoms. It has been reported that, for the FeNiB amorphous alloy, the exchange parameter follows the order of J_Fe–Ni_ > J_Fe–Fe_ > J_Ni–Ni_ [38]. On the other hand, for the FeCoB amorphous alloy, the trend is J_Fe–Co_ > J_Co–Co_ > J_Fe–Fe_ [39]. This shows that the addition of Co or Ni atoms enhances the magnetic interaction between different neighboring magnetic atoms. Therefore, Co or Ni atoms can induce a larger magnetic moment in the surrounding Fe atoms, but their own magnetic moment does not vary significantly.

## 4. Conclusions

In summary, the local atomic structure and electronic properties of Fe_80-x_TM_x_B_20_ (x = 0, 10, 20, 30, and 40 at.%; TM: Co, Ni) amorphous alloys were studied by means of ab initio molecular dynamics simulations. The influence of the increase in the Co or Ni content on the structure and magnetic properties of Fe-based amorphous alloys was discussed. For Fe_80−x_Co_x_B_20_ (x = 0, 10, 20, 30, and 40 at.%) amorphous alloys, the probability of obtaining a complete icosahedron is the highest at x = 10 at.%, which leads to a much more local structural symmetry enhancement, thus increasing the exchange splitting energy of the alloy and finally leading to the maximum magnetic moment. The magnetic moment of Fe_80−x_Co_x_B_20_ amorphous alloys gradually decreases due to the dilution effect for x > 10 at.%. For Fe_80−x_Ni_x_B_20_ (x = 0, 10, 20, 30, and 40 at.%) amorphous alloys, the magnetic moment decreases continuously with the increase in the Ni content due to the dilution effect. For the two types of alloys, the trends of the calculated magnetic moments as a function of the Co or Ni composition are all in good agreement with the experimental observations.

The exchange interaction between the adjacent magnetic atoms in Fe-based amorphous alloys is closely related to the doping elements. With the addition of Co or Ni, the magnetic interaction in the amorphous alloy changes. The Co (Ni) atoms can induce a larger magnetic moment in the surrounding Fe atoms; however, the magnetic moment of the Co or Ni atoms remains almost unchanged. This is also one of the reasons why the magnetic moment of the FeCoB amorphous alloy increases with the increase in the Co content in the case of a small amount of Co doping.

## Figures and Tables

**Table 1:** Valence electron distribution of each element for different contents of Fe_80−x_Co_x_B_20_ and Fe_80−x_Ni_x_B_20_ (x = 0, 10, 20, 30, and 40 at.%) amorphous alloys.

x	Average (diff)	Fe_80−x_Co_x_B_20_			Fe_80−x_Ni_x_B_20_		
		Fe (3*d*^6^4*s*^2^)	Co (3*d*^7^4*s*^2^)	B (2*s*^2^2*p*^1^)	Fe (3*d*^6^4*s*^2^)	Ni (3*d*^8^4*s*^2^)	B (2*s*^2^2*p*^1^)
0	Average (diff)	7.88 (−0.12)	/	3.46 (0.46)	7.88 (−0.12)	/	3.46 (0.46)
10	Average (diff)	7.863 (−0.137)	9.04 (0.04)	3.46 (0.46)	7.854 (−0.146)	10.13 (0.13)	3.44 (0.44)
20	Average (diff)	7.836 (−0.164)	9.05 (0.05)	3.44 (0.44)	7.828 (−0.172)	10.11 (0.11)	3.41 (0.41)
30	Average (diff)	7.818 (−0.182)	9.04 (0.04)	3.4 (0.4)	7.789 (−0.211)	10.11 (0.11)	3.36 (0.36)
40	Average (diff)	7.8 (−0.2)	9.01 (0.01)	3.38 (0.38)	7.754 (−0.246)	10.08 (0.08)	3.32 (0.32)

## Data Availability

Data is contained within the article.
